# Development of a Self-Emulsifying Drug Delivery System for Optimized Topical Delivery of Clofazimine

**DOI:** 10.3390/pharmaceutics12060523

**Published:** 2020-06-08

**Authors:** Daniélle van Staden, Jeanetta du Plessis, Joe Viljoen

**Affiliations:** Faculty of Health Sciences, Centre of Excellence for Pharmaceutical Sciences (PharmacenTM), Building G16, North-West University, 11 Hoffman Street, Potchefstroom, North-West Province 2520, South Africa; dvanstaden711@gmail.com (D.v.S.); Jeanetta.DuPlessis@nwu.ac.za (J.d.P.)

**Keywords:** topical delivery, self-emulsifying drug delivery system (SEDDS), clofazimine, penetration enhancers, pseudo-ternary phase diagrams

## Abstract

A quality-by-design and characterization approach was followed to ensure development of self-emulsifying drug delivery systems (SEDDSs) destined for topical delivery of the highly lipophilic clofazimine. Solubility and water-titration experiments identified spontaneous emulsification capacity of different excipient combinations and clofazimine. After identifying self-emulsification regions, check-point formulations were selected within the self-emulsification region by considering characteristics required to achieve optimized topical drug delivery. Check-point formulations, able to withstand phase separation after 24 h at an ambient temperature, were subjected to characterization studies. Experiments involved droplet size evaluation; size distribution; zeta-potential; self-emulsification time and efficacy; viscosity and pH measurement; cloud point assessment; and thermodynamic stability studies. SEDDSs with favorable properties, i.e., topical drug delivery, were subjected to dermal diffusion studies. Successful in vitro topical clofazimine delivery was observed. Olive oil facilitated the highest topical delivery of clofazimine probably due to increased oleic acid levels that enhanced stratum corneum lipid disruption, followed by improved dermal clofazimine delivery. Finally, isothermal microcalometric experiments studied the compatibility of excipients. Potential interactions were depicted between argan oil and clofazimine as well as between Span^®^60 and argan-, macadamia- and olive oil, respectively. However, despite some mundane incompatibilities, successful development of topical SEDDSs achieved enhanced topical clofazimine delivery.

## 1. Introduction

Resistance against anti-tubercular (A-TB) treatment regimens is considered a global health threat of the modern-age [1]. An estimated 500,000 individuals are newly infected with multi-drug resistant Tuberculosis (MDR-TB) each year [2] Additional to increased incidences of MDR-TB, rare extra-pulmonary manifestations of Tuberculosis (TB), such as cutaneous Tuberculosis (CTB), are escalating due to A-TB drug resistance [3]. A-TB treatment is extremely extensive as well as costly [1]. Moreover, presently no topical treatment is available to aid in CTB [3]. Therefore, CTB patients are subjected to general A-TB regimens [4]. These regimens rely on drugs with both reasonable and serious adverse effect profiles [1]. However, the development of a topically administered dosage form that assists in CTB therapy, can eliminate gastro-intestinal adverse effects by circumventing liver metabolism [5]. Similarly, topical A-TB drug delivery will enable treatment at the affected site without interference with oral A-TB management since most patients infected with CTB also suffer from pulmonary TB disease [4,6].

The focus of A-TB research has shifted towards repurposing known drug entities, with relatively safe adverse effect profiles to form part of new regimens for the resolution of decreasing treatment time and improving efficacy against TB [7]. Clofazimine is currently listed by the World Health Organization as a group 5 drug providing relief from MDR-TB [8] as it is a favorable candidate which has exhibited in vivo and in vitro efficacy against strains of MDR-TB with limited toxicity [9,10]. This repurposed riminophenazine antibiotic agent is furthermore administered as part of the triple regimen employed during treatment of multibacillary leprosy [8]. Its significantly high lipophilicity and its redox potential establish anti-microbial effectiveness by means of oxidation of reduced clofazimine followed by the formation of reactive oxygen species [3]. Additionally, possible synergism between clofazimine and A-TB drugs, including capreomycin and moxifloxacin, has been reported [8,11]. However, its physicochemical properties are not particularly suitable for dermal drug delivery with an aqueous solubility of 0.225 mg/L (practically insoluble) and Log P of 7.66 to name a few [3,12]. Nonetheless, the development of an efficacious topical drug delivery system could potentially enable optimized topical delivery of clofazimine.

The concept of self-emulsifying drug delivery systems (SEDDSs) was pioneered during the 1960s when components of poor aqueous solubility were incorporated into mixtures of lipophilic and hydrophilic excipients to achieve enhanced solubility of lipophilic substances [13]. However, Pouton [13,14,15,16] only suggested utilizing SEDDSs in 1985 as the first concrete solution towards improving lipophilic drug delivery via the oral route of administration. Despite several decades devoted to SEDDS development for various routes of administration, namely: oral, rectal, vaginal, ocular and nasal; the dermal route has remained relatively untouched [17,18,19,20,21,22,23]. The formidable barrier provided by the outermost skin layer can possibly be conquered by SEDDSs as these isotropic, thermodynamically stable mixtures, comprising oil, surface active agents and water, have the potential capacity to facilitate entry of drugs into underlying skin layers [24,25]. Modification of stratum corneum (SC) lipid arrangement can be achieved by natural oils included in topical SEDDSs [26,27,28,29]. Additionally, oils are able to assist in solubilizing highly lipophilic clofazimine as only solubilized drug particles can partition into the SC [30,31].

Five natural oils were selected as potential lipophilic components in the development of topical SEDDS. Argan, avocado, coconut, macadamia and olive oil were chosen due to the unique fatty acid composition of each natural oil. Fatty acid profiles of natural oils can significantly influence dermal clofazimine delivery since drugs as lipophilic as clofazimine generally tend to accumulate in the fatty environment of the SC instead of partitioning into the underlying, hydrophilic epidermis [32,33]. Skin penetration enhancers, such as natural oils, can facilitate SC lipid disruption by mechanisms such as fluidization and loosening the ordered SC lipid structure to allow improved partitioning of clofazimine into underlying skin layers in order to achieve successful topical delivery [29]. Briefly, avocado oil was considered due to its increased palmitic acid content, which portrays significant skin penetration enhancing effects [34]. Argan oil was chosen due to its elevated levels of stearic- and linoleic acid [35,36]. Conversely, coconut oil is a rich source of lauric and myristic acid that contribute to the increased saturated fatty acid content of this natural oil [34]. Macadamia oil can potentially provide insight into the penetration enhancement effects of palmitoleic- and linolenic acid [34]. Last, olive oil was selected to enable investigation of the effect of increased levels of oleic acid on skin penetration enhancement [34]. The fatty acid composition of these natural oils is presented in the Supplementary Material.

The inclusion of surface active agents can maintain formulation stability while facilitating increased disruption of SC lipids [29]. According to literature the surface active agent employed during formulation of SEDDSs should have a HLB value of ≥12 in order to provide sufficient phase stabilization [30,37]. Therefore, Tween^®^80 (HLB value = 15) was chosen as the designated surfactant for this study. Additionally, Tween^®^80 is often employed as a surface active agent during formulation of topical/transdermal products due to its non-ionic nature that leads to a decreased risk of skin irritation [38]. Span^®^60 was subsequently selected as co-surfactant for the purpose of improving flexibility of the interfacial film established by the surfactant, contributing towards an even more stable formulation [30,37]. Moreover, the inclusion of co-surfactants establishes finer droplet formation and will therefore also render improved solubility of the incorporated clofazimine [30] Additionally, the combination of Tween^®^80 and Span^®^60 is frequently utilized during development of formulations destined for dermal application [39].

This explorative research aimed at developing topical clofazimine SEDDSs aiding in CTB as well as establishing criteria for these systems since SEDDSs provide simplified techniques suitable for industrial upscaling together with improved drug solubility [24,25] which has not yet been investigated for topical drug delivery.

## 2. Materials and Methods

### 2.1. Materials

Clofazimine was generously donated by Cipla Pty Ltd. (Mumbai, India). Argan, avocado, coconut, macadamia and olive oil were purchased from Scatters Oils (Johannesburg, RSA). Tween^®^80 and Span^®^60 were obtained from Associated Chemical Enterprises (Pty) Ltd. (Johannesburg, RSA) and Sigma-Aldrich Chemistry GmbH (Steinheim, Germany). Distilled water was attained through a Rephile Bioscience Ltd. system (Boston, Massachusetts (MA), USA).

### 2.2. Pre-Formulation Studies

#### 2.2.1. Solubility

Excess clofazimine was added to 5 mL of each oil tested and vortexed for 2 min, where after the samples were shaken in a water bath (32 ± 0.5 °C) for 48 h. Samples were centrifuged at 3000 rpm for 15 min at 22 °C. Next, 1 mL supernatant was removed from each sample and diluted to 20 mL with methanol. Analysis (UV wavelength: 284 nm) was done utilizing an Agilent^®^ 1100 HPLC system (25 ± 0.5 °C) equipped with an Agilent^®^ 1100 pump, UV-detector, and auto-sampler injection mechanism (Agilent Technologies, Palo Alto, California (CA), USA). Chemstation Rev. A10.02 software (Agilent Technologies, Palo Alto, California (CA), USA) was employed for data acquisition and samples were analyzed in triplicate [40,41,42,43].

#### 2.2.2. Pseudo-Ternary Phase Diagrams

Employing the water titration method, pseudo-ternary phase diagrams were constructed, where one of the oils, water and surfactant phase (Tween^®^80 and Span^®^60) formed the triplot. The surfactant phase was fixed at a 1:1 concentration ratio as literature concluded it to form more stable SEDDSs, whereas higher ratios enlarge the emulsion range, but facilitate decreased stability that could cause precipitation of an incorporated drug [44,45]. Clofazimine concentrations (% w/w) varied according to its solubility in each oil [40]. Specifically, 0.6% was incorporated into avocado (AVO) oil preparations, whereas 0.2% was added to argan- (ARG) or coconut (CCT) oil preparations. Likewise, 0.1% was included in macadamia- (MAC) or olive (OLV) oil formulations. The surfactant phase and selected oils were prepared in fixed ratios (9:1, 8:2, 7:3, 6:4, 5:5, 4:6, 3:7, 2:8 and 1:9) while water (varying component) was added in a dropwise fashion at ambient temperature [40]. The point at which the preparations turned turbid is considered the end-point; and these points were plotted implementing Triplot software version 4.1.2 to construct pseudo-ternary phase diagrams that identified the area of spontaneous emulsification for each oil.

### 2.3. Preparation of Topical SEDDSs

The surfactant phase was prepared by continuous heating and stirring for 25 min. Clofazimine was dissolved in each oil phase while subjected to sonication for 2 min, employing a UP400St (400W, 24 kHz) Hielscher’s digital ultrasonic device (Hielscher Ultrasonics, Teltow, Germany). Subsequently, each oil phase was added to the surfactant phase with continuous stirring for an additional 25 min. Small increments of water were added with waiting periods in-between until the full predetermined quantity was included. The SEDDSs were left to cool before storage at room temperature (25 ± 0.5 °C) for 24 h; followed by visual observation of phase separation to identify any indication of formulation instability [46,47,48]. SEDDSs were exposed to increased temperatures during production. This is not traditional, since low inert energy of excipients normally facilitates spontaneous emulsification once exposed to gentle agitation. However, heating during water titration followed by temperature stabilization at 25 °C has been reported to render spontaneous self-emulsification, especially in systems that should conquer high kinetic barriers [46].

### 2.4. Characterization of Topical SEDDSs

#### 2.4.1. Droplet Size, Zeta-Potential and Size Distribution

Droplet size, zeta-potential and size distribution were assessed by means of dynamic light scattering performed by a Zetasizer Nano^®^ ZS (Malvern^®^ Instruments Ltd., Worcestershire, UK) at 25 °C.

#### 2.4.2. Robustness to Dilution

Topical SEDDSs were diluted 100-fold with distilled water or phosphate buffer solution (PBS) comprising different pH values. The pH of PBS was adjusted to 5, similar to skin surface pH; and 7.4 to resemble change in pH prior to partitioning into the epidermis [49]. These dilutions were visually inspected for phase separation after storage at 25 °C for 24 h [43].

#### 2.4.3. Efficacy and Self-Emulsification Time

A type II Distek 2500 dissolution system apparatus (Distek, North Brunswick, New Jersey (NJ), USA) was operated; where 1 mL SEDDSs was added to 100 mL distilled water and mildly agitated with a paddle speed of 50 rpm at 32 ± 0.5 °C (skin surface temperature). The time required by individual SEDDSs to transpire into homogenous dispersions was noted and the efficacy of spontaneous emulsification graded according to Table 1 [43,50].

#### 2.4.4. Viscosity and pH

A Brookfield^®^ Viscometer model DV-II+ (Brookfield Engineering Laboratories, Inc., Stoughton, Massachusetts (MA), USA), attached to a circulating water bath, equipped with a Brookfield^®^ temperature controller, was employed. The temperature of the water jacket was sustained at 25 ± 0.5 °C [51] and different spindles (SC4-34 LV, SC4-25 LV, T-bar F LV and T-bar E LV) were used at 20 rpms where torque values of approximately 20% were maintained. The pH of selected SEDDSs was assessed using a Mettler^®^ Toledo pH meter with a Mettler^®^ Toledo Inlab^®^ 410 NTC electrode 9823 (Mettler^®^ Toledo International Inc., Columbus, Ohio (OH), USA). Calibration of the electrode was executed at a pH of 4, 7 and 10 prior to measurement [52].

#### 2.4.5. Cloud Point

Dilutions with distilled water (1:100) of individual SEDDSs were positioned in a water bath at a starting temperature of 25 °C. The initial temperature was slowly increased at 2 °C/min until a turbid appearance of the formulations was visually observed upon dehydration of excipients [53].

#### 2.4.6. Thermodynamic Stability Studies

SEDDSs were exposed to varying temperatures (i.e., heated and cooled) during six cycles of approximately 4 °C and 45 °C, not exceeding 48 h. Each tested SEDDS was visually inspected for any possible phase separation or drug precipitation [43]. Hereafter, the SEDDSs were centrifuged at 3500 rpm for 30 min and again visually assessed for any indications of instability, e.g., phase separation, cracking and/or creaming [54].

### 2.5. Topical Delivery

#### 2.5.1. Encapsulation Efficiency

To establish the encapsulation efficiency (%EE), the centrifugation separation method was employed utilizing an Eppendorf^®^ 5804 R centrifuge equipped with an A-4-44 rotor (Merck, Modderfontein, RSA). A sample (1 mL) of each selected SEDDSs was centrifuged at 1500 g for 20 min for the unentrapped clofazimine to form a pellet in the tube. The supernatant was collected and transferred into HPLC vials, followed by diluting with a specific volume of mobile phase and analysis by means of HPLC. The unentrapped clofazimine was subtracted from the initial added amount and a percentage calculated, signifying the %EE [3].

#### 2.5.2. Drug Release Experiments

Drug release studies (6 h) were performed prior to skin diffusion experiments in order to determine whether the selcted SEDDS formulations would release clofazimine. These tests were conducted in the same manner as the skin diffusion experiments (following section), although polytetrafluoroethylene membranes were employed instead of excised human skin. PBS from the receptor compartments were extracted every hour for 6 h [28].

#### 2.5.3. Skin Preparation

Full-thickness Caucasian female skin, donated by anonymous patients that underwent abdominoplastic surgery, was employed during dermal diffusion experiments. Ethical endorsement concerning skin procurement and preparation was granted by the Ethics Committee of the North-West University, RSA (ethics number: NWU-00111-17-A1-07).

Skin was immediately stored at −20 °C at the bio-safety laboratory for no longer than 6 months. Preceding investigation, skin was allowed to thaw at 25 °C and visually examined for abnormalities that might affect dermal diffusion, including striae distensae. The skin was cut into pieces of approximately 2 cm × 4 cm and 400 m thick with a Zimmer^®^ electric dermatome, model 8821 (Zimmer^®^ Ltd., Swindon, Wiltshire, UK). These harvested pieces were placed onto Whatman^®^ filter paper, cut into circles and covered in aluminum foil, after which it was stored at −20 °C until testing within 24 h [3].

#### 2.5.4. Skin Diffusion Studies

Franz diffusion cells (n = 10) were implemented to conduct in-vitro dermal diffusion experiments for each selected SEDDS. Skin circles were mounted between the receptor- and donor compartment; where the receptor phase consisted of PBS (pH 7.4) that was continuously stirred with a magnetic stirrer at 720 rpm. It is stated that drugs with an aqueous solubility ≤0.1 mg/mL acquire the inclusion additional solubilisers in the receptor compartment for the purpose of providing a solvent that solubilises the lipophilic drug as a drug must be in solution in order to be detected by means of HPLC analysis [55,56]. For this reason, analytical grade ethanol was included in the receptor phase in a 9:1 ratio (PBS:ethanol) for the purpose of improving the solubility of clofazimine during the conduction of dermal diffusion studies. The solubility of clofazimine in this mixture was determined in triplicate prior to dermal diffusion experiments to ensure HPLC detectability, if the receptor phase were to be reached. An average clofazimine solubility of 0.37 ± 0.78 mg/mL was obtained, which was considered sufficient to detect any transdermal clofazimine diffusion as well as able to quantify the data, since the limit of quantification was established as 0.0003 mg/mL (%RSD: 1.60%). Stability results furthermore rendered a yield of 100.01 ± 0.85% over a period of 24 h during analytical method validation development.

The donor compartment contained 1 mL of a selected SEDDS and was covered with Parafilm^®^ in order to avoid vehicle metamorphosis. Franz cells were positioned in a water bath retained at 37 °C beforehand; the investigation was initiated approximately 5 min post experimental setup; the receptor phase was completely extracted after 12 h. Samples were analyzed in triplicate utilizing a validated HPLC method [3].

#### 2.5.5. Tape Stripping

Skin circles were removed from the Franz cells directly after diffusion experiments and secured onto a board covered with Whatman^®^ filter paper. Excess SEDDSs were wiped from skin surfaces and clear Scotch^®^ magic tape strips were applied to remove the SC-epidermis fraction. The first two tape strips were discarded (cleaning) and the following 15 were deposited into a polytop filled with 5 mL analytical grade ethanol and stored (2–8 °C) for about 8 h. The residual epidermis-dermis of the diffusion region was cut into small pieces and placed into individual polytops filled with 5 mL analytical grade ethanol prior to storage at 2–8 °C for approximately 8 h. Next, samples were filtered (45 μm), transferred into HPLC vials and subsequently analyzed in triplicate [3].

### 2.6. Isothermal Calorimetry

The compatibility of excipients was determined according to a previously published method [3] utilizing a 2277 Thermal Activity Monitor, TAMIII, (TA Instruments, New Castle, Delaware (DE), USA) equipped with an oil bath with a stability of ±100 μK over 24 h. The temperature was maintained at 40 °C and 100 mg samples tested. Heat flow was measured for individual components to obtain a theoretical response (i.e., baseline) which was followed by comparison of the theoretical response to the measured calometric output to determine compatibility. If the theoretical response drastically fluctuates from the measured calometric output, interactions between excipients are considered plausible. Generally, a change in heat flow that exceeds 10 μW/g, with additional slopes observed on heat flow graphs, signifies potential incompatibility.

## 3. Results and Discussion

### 3.1. Pre-Formulation and Characterization

#### 3.1.1. Solubility Studies

Normally, oils are included in SEDDSs to enhance and maintain solubility of highly lipophilic drugs as they solubilize drug particles which are then able to partition into the outermost skin layer [30,31]. Moreover, oils provide reversible alteration of SC lipids that facilitate dermal penetration enhancement [26,27,28,29]. Literature indicated the aqueous solubility of clofazimine as 0.225 mg/L (2.25 × 10^−4^ mg/mL) [12]. Thus, solubilizing clofazimine in any of the selected oils significantly improved its solubility as seen in Table 2; indicating that a higher fraction of solubilized clofazimine will probably be able to penetrate the skin.

#### 3.1.2. Pseudo-Ternary Phase Diagrams and Topical SEDDSs Preparation

**Figure 1:**
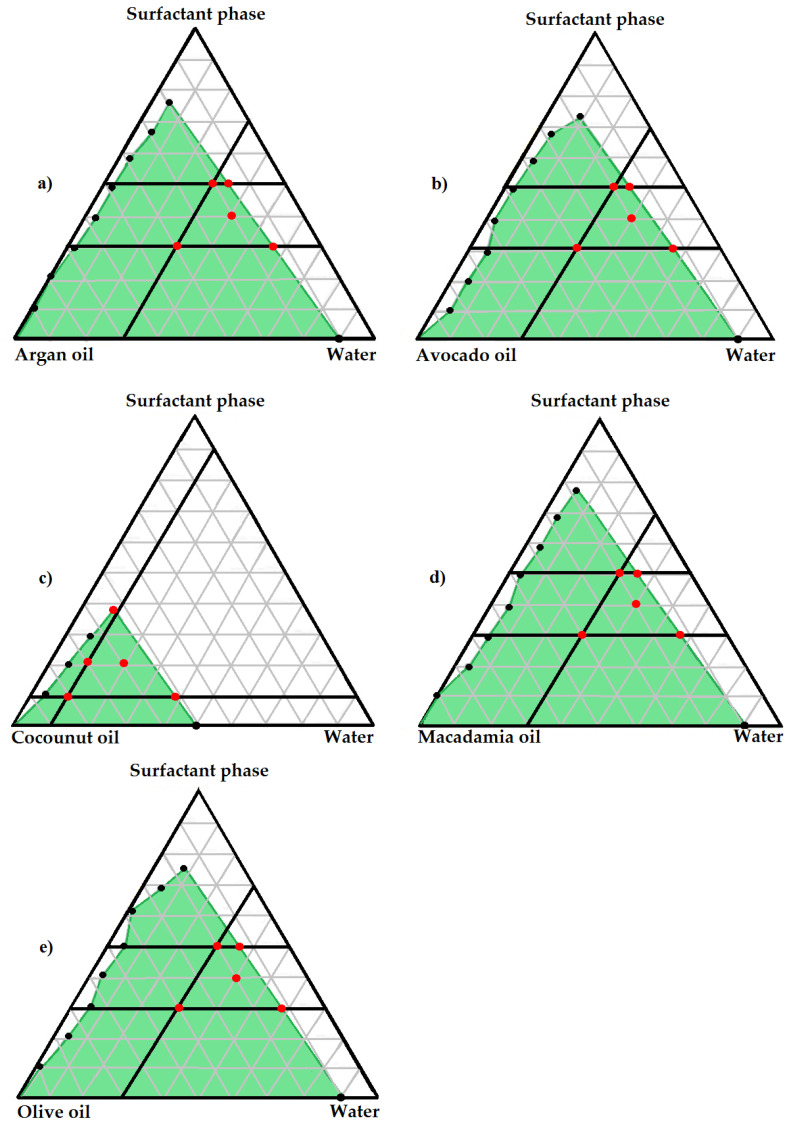
Phase diagram indicating check-point formulations for (**a**) argan oil, clofazimine, surfactant phase and water; (**b**) avocado oil, clofazimine, surfactant phase and water; (**c**) coconut oil, clofazimine, surfactant phase and water; (**d**) macadamia oil, clofazimine, surfactant phase and water; (**e**) olive oil, clofazimine, surfactant phase and water.

Pseudo-ternary phase diagrams provide assistance in identifying the self-dispersability potential of SEDDSs [30]. Moreover, these diagrams schematically represent the concentration range of the utilized excipients that can facilitate self-emulsification when incorporated in combination [57]. It is evident from Figure 1 that a notably large region exists on the pseudo-ternary phase diagrams for ARG, AVO, MAC and OLV SEDDSs where possible spontaneous emulsification can transpire. Following, a specific area within the self-emulsification region of each selected oil was identified that was deemed suitable for topical application.

This zone was selected by eliminating areas that are known to specifically present unfavorable properties for topical drug delivery. For example, only regions containing a surfactant phase ratio of ≤5 were considered, as an increased surfactant concentration is known to cause skin irritation [58,59]. Likewise, micelles obtained in water-rich areas [60], are unwanted structures due to their rigidity and reduced deformability, rendering poor dermal drug delivery [61,62]. Hence, formulations deliberated suitable should not exceed a water content ratio of 7. Furthermore, high oily content areas tend to produce reverse micelles [60], defying the purpose of developing topical SEDDSs. Therefore, self-emulsification regions exceeding an oil ratio of 7 were also excluded. A possible five formulations were consequently prepared from each individual oil’s self-emulsification region (Supplementary Material, Section B), i.e., two points at the top, two at the bottom, and one in the center of the identified region on the pseudo-ternary phase diagrams (Figure 1). The self-emulsification area on the pseudo-ternary phase diagram of CCT rendered a markedly smaller range comparatively. Nonetheless, five possible formulations were selected from this region as similarly as possible to the selection method utilized for the other oils.

Visual inspection of the topical SEDDSs, retained at ambient temperature for 24 h, identified phase separation in ARG2, ARG4, AVO1, MAC4, OLV4 as well as all of the prepared CCT SEDDSs (Supplementary Material, Section B). These SEDDS formulations were considered unfavorable for topical clofazimine delivery as they were deliberated unstable formulations and were subsequently excluded from further analysis. The remaining SEDDSs (Table 3) were deemed suitable for further characterization experiments to generate profiles so as to establish which SEDDSs are most suitable for dermal drug delivery, prior to conducting dermal diffusion studies.

#### 3.1.3. Droplet Size, Zeta-Potential and Size Distribution

Drug delivery facilitated by SEDDSs are essentially influenced by droplet size, droplet size distribution and zeta-potential [41,63,64]. Size characterization is considered one of the most insightful experiments performed during the development of SEDDSs as size influences not only drug release, but also the stability of the SEDDS formulations [65]. It has been reported that reduced droplet size can contribute towards rapid and significantly increased drug permeation during dermal drug delivery [66]. In addition, smaller droplets portray a decreased tendency towards emulsion instability such as cohesion. Most topical SEDDSs tested (Table 3 where SEDDSs that did not meet the specifications are in bold and highlighted) fell within the micro-sized range, i.e., 100–250 nm. Favorably, ARG1, AVO2 and AVO5 could be classified as nano-SEDDSs (˂100 nm) [30]. However, ARG5, AVO3, MAC5, OLV1 and OLV3 demonstrated droplet sizes >250 nm and are therefore not deliberated ideal for dermal diffusion studies [66].

Uniform size distribution of droplets within dispersions is additionally indicative of formulation stability and is scrutinized through the polydispersity index (PDI) [66]. However, no fixed PDI-criteria have yet been established for dermal drug delivery, except for lipid based carrier systems (PDI < 0.3) and polymer-based nanoparticles (PDI ≤ 0.2) developed specifically for transdermal drug delivery. Moreover, a PDI exceeding the generally accepted pharmaceutical range of 0.05–0.7, can designate that microscopic techniques must be employed for size characterization rather than dynamic light scattering as it could possibly mistakenly identify many small particles clustered together as single large particles [67]. In this study only PDI was evaluated (no other microscopic techniques were employed) as there are no criteria specifically set for topical and transdermal SEDDSs yet. The other tests conducted, e.g., droplet size analysis, assisted with further elucidation, and SEDDSs that obtained a PDI of 1 (ARG5, AVO3, AVO4, MAC5, OLV1, OLV2 and OLV3) were excluded from further analysis (Table 3, where SEDDSs failing the criteria are highlighted and in bold).

It is known that increasingly negative or positive zeta-potential values (i.e., >30 mV or ˂−30 mV) signify increased electrostatic repulsion between droplets, and are therefore considered favorable as coagulation is circumvented [68]. Nevertheless, a minute deviation is allowed as research recognized that emulsions stabilized by both steric and combined electrostatic forces, as enabled by Tween^®^80, with a minimum zeta-potential value of −20 mV, can be contemplated acceptable [69,70]. The negatively charged zeta-potential values obtained (Table 3) are initiated by the presence of free fatty acids within the oil phase [71]. However, as the net charge of the skin is negative, a positively charged formulation should theoretically facilitate increased affinity between the applied formulation and skin [72,73]. Contrarily, free fatty acids are skin penetration enhancers that function by disruption and fluidization of SC lipids to enhance dermal drug delivery [26,27,28,29]. Therefore, negatively charged SEDDSs can still potentially facilitate dermal drug delivery, but just in a slower fashion compared to positively charged formulations [72]. Accordingly, all SEDDSs tested, complied with the criteria set for zeta-potential, where overall, the SEDDSs comprising AVO are regarded most stable (average zeta-potential: −37.35 mV). Interestingly, a co-relation probably exists between the pH of SEDDS and zeta-potential measurements as AVO SEDDS portrayed decreased pH measurements with increasingly negative zeta-potential values.

#### 3.1.4. Robustness to Dilution

It was accepted during visual observation of diluted topical SEDDSs that ARG5, AVO5, OLV2 and OLV3 revealed complete phase separation upon dilution with distilled water and PBS comprising different pH values. Thus, these formulations were deliberated unsuitable for dermal drug delivery. Although ARG3, AVO2, MAC1 and OLV1 displayed robustness towards exposure to different pH environments, but failed to withstand phase separation upon dilution with distilled water, they were still considered suitable for further analysis. This decision was based on the assumption that stability of these formulations will not be influenced while diffusing through different skin layers, as these layers have similar pH environments to the different PBS utilized during this experiment.

Furthermore, these formulations were diluted 100-fold according to the criteria set for oral SEDDSs [43], as no benchmark has been developed for topical/transdermal SEDDSs. However, exposure to large volumes of water on the skin surface will probably not transpire as sweat is the most noteworthy fluid that can influence stability of topically applied SEDDSs. The sweat rate of healthy individuals is between 500–700 mL daily over the entire body surface [74]. Thus, the possibility of exposing topically applied SEDDSs to a similar fluid volume of 99 mL, utilized to prepare these dilutions, on a single region of the body, is considered highly unlikely. For these reasons it was deliberated that the robustness to dilution test for SEDDSs is specifically suited for oral SEDDSs as these systems are administered with a glass of water and must remain stable upon further exposure to fluids within the gastro-intestinal tract [30,75]. When considering topical/transdermal drug delivery characterization, we established that the focus in this case should rather be on the ability of SEDDSs to withstand phase separation when exposed to different pH environments rather than the volume to which the SEDDSs were exposed to. Moreover, the robustness to dilution criteria specifically for topical/transdermal SEDDSs should be refined according to various factors that need to be considered; for example, volume of SEDDS to be applied, properties of affected area, area of exposure, etc. These criteria thus need to be researched in more detail. For these reasons, ARG5, AVO5, OLV2 and OLV3 were eliminated from further investigation, since these formulations also failed other characterization assessments, including robustness to dilution at different pH values.

#### 3.1.5. Efficacy and Self-Emulsification Time

Efficacy of self-emulsification is also referred to as dispersability assessment [76,77,78]. Swift emulsification is considered highly favorable, if observed with SEDDSs intended for oral administration, as spontaneous emulsification is identified as the rate limiting step that must occur before successful absorption can ensue [78]. On the other hand, during topical drug delivery, diffusion through the lipophilic SC marks the rate limiting step for most drugs [29,79]. Thus, prolonged contact time between SEDDSs and skin can determine if sufficient diffusion of the drug across the SC can be achieved [19,80].

Considering the grading system for emulsification behavior exhibited by SEDDSs upon dilution as displayed in Table 1, SEDDSs that obtained a C- or D-grading could be deemed promising for dermal clofazimine delivery. However, SEDDSs that demonstrated poor emulsification properties, with consequent E-grading, were considered unsuitable as slow emulsification is favorable, but complete inability to self-emulsify is undesired. Moreover, SEDDSs that rendered rapid emulsification (A- or B-grading) were also reasoned inapt as these SEDDSs can be easily washed away once exposed to sweat or external water. Therefore, rapid spontaneous emulsification is suggestive of decreased occlusivity that sequentially reduces topical clofazimine delivery [80,81]. Hence, MAC5 (Table 3, in bold and highlighted) was discarded in terms of dermal drug delivery due to a received B-grading (Table 1).

Self-emulsification time of individual SEDDSs illustrates the free energy needed to enable self-emulsification that can be influenced by the energy decreasing capabilities of surface active agents, which dictate entropy of formulations [82]. Literature confirmed that spontaneous emulsification can either occur swiftly or be prolonged, depending on the presence of kinetic barriers between excipients included in SEDDSs [46]. What is more, kinetic barriers can be overcome through applying heat or mild agitation [46]. Thus, definite kinetic barriers are present as clofazimine SEDDSs required exposure to heightened temperatures, followed by cooling afterwards, to enable spontaneous emulsification [46]. Overall, ARG SEDDSs required longer intervals to achieve self-emulsification that possibly shows increased kinetic barriers between the excipients of these SEDDSs (Table 3).

#### 3.1.6. Viscosity and pH

Viscosity refers to internal friction of a fluid that can elicit an impact on flow resistance and spontaneous emulsification [83,84]. Incorporated natural oils have a definite influence on the viscosity of SEDDSs considering that ARG3, AVO3, MAC3 and OLV3 comprise the same exact excipient ratios (Supplementary Material Section B); however, noteworthy differences in their viscosity exist (Table 3). These viscosity values, according to the oil-type included, could be ranked as: OLV > ARG > MAC > AVO. Prolonged self-emulsification times were depicted by formulations with enhanced viscosity (ARG3 and OLV3) compared to self-emulsification times (Table 3) exhibited by SEDDSs with lower viscosity values (MAC3 and AVO3). Thus, suggesting a potential direct correlation between the ease of spontaneous emulsification and viscosity. Additionally, a general trend was observed with the different MAC and OLV SEDDS formulations. As the average droplet size of these SEDDSs increased, a decrease in their viscosity was depicted. It has been established that the smaller the droplet size of a formulation, the higher dermal drug delivery will be; whereas formulations displaying increased viscosity values will normally portray higher occlusivity. Therefore, in this case, MAC and OLV SEDDS formulations comprising smaller droplet sizes will probably demonstrate enhanced dermal drug delivery due to their smaller droplet sizes as well as these formulations having increased occlusivity. No clear correlation could be obtained for the ARG or AVO SEDDS formulations.

In view of the pH of SEDDSs for dermal drug delivery, a range deemed suitable is 5.0–9.0 [85]. However, an optimal pH would closely resemble the natural pH of skin and will thus range between 4.5 and 5.0 [49]. Most formulations depicted values of 5.0–9.0, except AVO3 that exhibited an unacceptable pH measurement of 3.82 (Table 3, indicated in bold and highlighted). Interestingly, overall, AVO SEDDSs portrayed pH measurements closely related to the natural pH of skin.

#### 3.1.7. Cloud Point

Cloud point specifies the temperature where SEDDSs are incapable of retaining their spontaneous emulsification properties [86]. This leads to erratic release of the incorporated drug and may further trigger irreversible phase separation [53], which is linked to dehydration of excipients once exposed to heightened temperatures [63]. ARG1, MAC1 and MAC3 (Table 3, specified in bold and highlighted) portrayed excipient dehydration below skin surface temperature (32 °C) rendering them unsuitable for dermal delivery [50].

#### 3.1.8. Thermodynamic Stability

Inclusion of surface active agents in SEDDSs cannot promise physical stability due to the complexation of emulsified systems sustained by surfactants. These excipients create interfacial tension gradients and provide modification to the breakup dynamics of droplets [87]. Hence, the physical stability of SEDDSs was investigated through thermodynamic stability experiments [43,54].

It is evident that ARG5, MAC1, OLV1 and OLV2 were unable to withstand environments of thermodynamic- and kinetic stress. Contrary, OLV3 exclusively portrayed instability once subjected to differing temperatures; whereas AVO5 was unable to remain stable upon centrifugation. Thus, these formulations that illustrated any instability once subjected to thermodynamic and/or kinetic stress conditions were deemed inapt for topical drug delivery.

### 3.2. Topical Clofazimine Delivery

Post characterization, only ARG3, AVO2, MAC2 and OLV5 SEDDSs were reasoned suitable candidates for topical clofazimine delivery. Subsequently the %EE was determined for each individual selected SEDDS. SEDDSs containing ARG, MAC or OLV as natural oil portrayed a %EE higher than 90%. Clofazimine entrapped either within MAC2 or OLV5 exhibited the highest %EE (both = 95.0%), whereas clofazimine in AVO2 rendered the lowest (70.0%) %EE. Overall, the following rank order could be established: MAC2 = OLV5 > ARG3 >>> AVO2. Interestingly, the ability to entrap clofazimine could not be linked to the oil- or water content of the SEDDS formulations as both MAC2 and AVO2 comprised the exact same ratios. Contrary, droplet size, PDI and zeta potential were found to be interrelated in this case. As the droplet size and PDI values increased and the zeta potential decreased; the %EE was enhanced. Clearly the larger the oil droplets (irrespective of the oil type) in which the clofazimine dissolved, and the less repulsive forces between these droplets, the more easily clofazimine was encapsulated.

Following, drug release studies utilising synthetic membranes were performed to confirm release of clofazimine from the selected SEDDSs prior to performing dermal diffusion experiments. Favourably, all of the SEDDS formulations tested, released detectible clofazimine concentrations as all of these SEDDSs exhibited some degree of diffusion through the membranes into the receptor compartments. Data obtained are expressed as a percentage of the initial quantity of clofazimine included in the finally selected SEDDS formulations as seen in Table 4.

The average percentage clofazimine released could be ranked as follows: OLV5 >>> AVO2 > MAC2 >> ARG3. Curiously, one must bear in mind that argan, avocado, macadamia and olive oil all contain oleic acid (Supplementary Material, Section A). However, each plant-based oil consists of a different oleic acid concentration, which may be classed from highest to lowest oleic acid concentration: olive oil >>> avocado oil >> macadamia oil > argan oil. Thus, the average percentage clofazimine was released according to the oleic acid content of the oils. However, it should be highlighted that topical SEDDSs utilized to conduct membrane release studies contained diverse clofazimine concentrations. These concentrations were determined according to the solubility of clofazimine in each plant-based oil. Significant differences (*p* < 0.001) between the cumulative clofazimine concentrations and release rates of the selected SEDDSs could be established, however, these differences were smaller between MAC2 and OLV5. Overall, SEDDSs containing oils high in oleic acid (monounsaturated fatty acid) concentrations (Supplementary Material, Section A) therefore released more clofazimine than those containing oils with relative high linoleic acid concentrations (polyunsaturated fatty acid, i.e., branched-chain fatty acids). Moreover, SEDDS formulations comprising oils high in stearic acid (saturated fatty acid) together with an almost equal mixture of monounsaturated fatty acids and polyunsaturated fatty acids, released less clofazimine.

To improve clofazimine detection and incorporate a therapeutic concentration, 2% w/w clofazimine was included in each of the selected SEDDS formulations (ARG3, AVO2, MAC2 and OLV5). Supersaturation is a relatively old, however, not-yet-optimized concept in terms of oral drug delivery [88]. Therefore, topical SEDDSs were prepared by an old-fashioned supersaturation technique of heating followed by cooling as described in literature [88]. As SEDDSs were heated during production of saturated and supersaturated SEDDSs, the heating and cooling process was considered a relatively normal procedure for these specific SEDDSs. Supersaturated SEDDSs were left at room temperature for 24 h after complete cooling in order to visually inspect the susceptibility of these dosage forms to fall victim to drug precipitation. Dermal diffusion studies are normally conducted over a 12 h period [3]. Hence, if topical SEDDSs are able to withstand drug precipitation for 24 h at room temperature, a stability for the period of 12 h is needed to perform dermal diffusion experiments should not pose problematic. Additionally, the delivery of supersaturated clofazimine concentrations at the skin surface are desired, as these supersaturated systems will probably enhance dermal flux [76,89] as a result of delayed nucleation and crystal growth that are enabled by SEDDSs due to enhanced kinetic- and thermodynamic inhibition of clofazimine precipitation [30].

**Figure 2:**
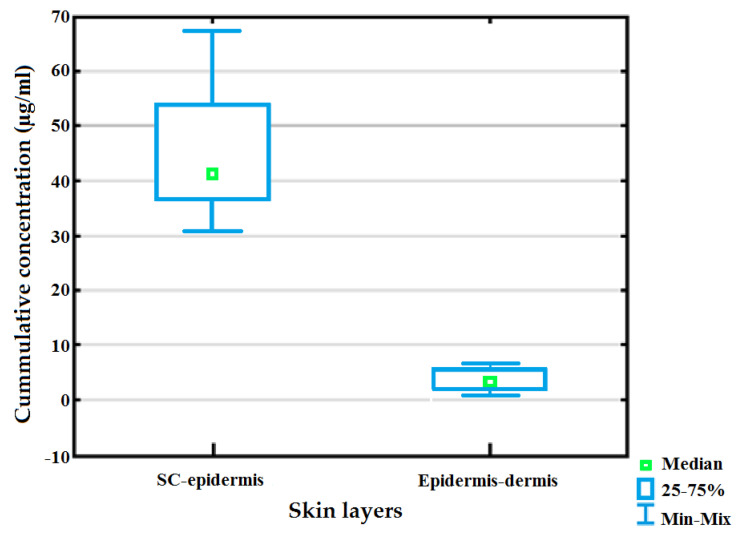
Cumulative clofazimine concentration delivered in different skin layers as achieved by MAC2 over a duration of 12 h.

No clofazimine was detected in the receptor phase post dermal diffusion regardless the SEDDS applied, thus favoring topical delivery and not distribution of clofazimine into the systemic circulation [5]. Patients diagnosed with CTB generally also suffer from pulmonary TB and will already be on systemic treatment [90]. For this reason, topical therapy should not interfere with systemic regimes as it may trigger additional side effects; therefore, this outcome is reasoned ideal. Moreover, clofazimine could only be detected in the epidermis-dermis layer after application of ARG3, AVO2 or OLV5; whereas it could be quantified in both the SC-epidermis and epidermis-dermis layers once MAC2 was tested. Clofazimine concentrations were statistically significantly (p < 0.001) increased in the SC-epidermis relative to the epidermis-dermis. A previous study specified that MAC comprises a high palmitoleic acid content that has preferred affinity for the epidermis as shown in Figure 2 [91]. This increased affinity is due to a higher molecular weight owing to a longer carbon backbone (>C14) that deduces mobility of fatty acids throughout different skin layers [92]. Moreover, MAC2 depicted the highest viscosity value of all of the SEDDSs subjected to dermal diffusion studies. The increased viscosity may possibly be responsible for this formulation portraying improved occlusivity, which in turn will enhance dermal drug delivery as seen with MAC2 where clofazimine was exclusively detected in the underlying skin-layers, i.e., the epidermis-dermis.

**Figure 3:**
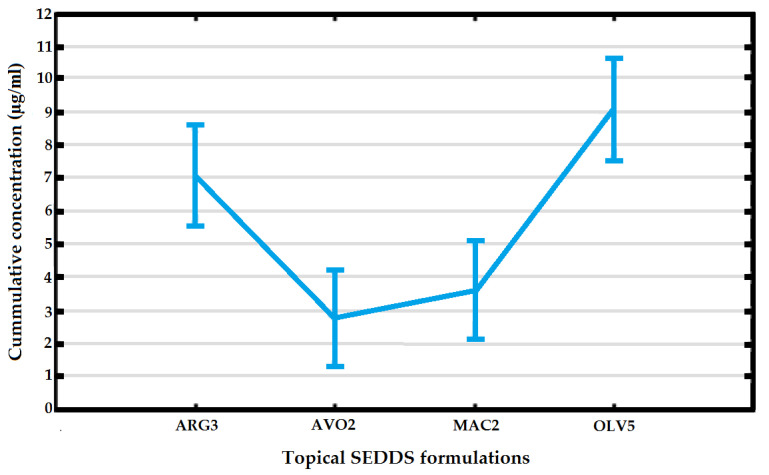
Cumulative clofazimine concentrations observed in epidermis-dermis.

Overall, OLV5 rendered the highest dermal cumulative clofazimine concentration, followed by ARG3 (Figure 3), which may be attributed to enhanced oleic acid content known for its penetration enhancement properties by means of SC disruption [93]. The natural lipid structure is disordered and the fluidization of these lipids occurs, which in turn facilitates improved dermal drug delivery [93,94]. Interestingly, speculation indicates that only the *cis* form of oleic acid is responsible for skin penetration enhancement, as established by the unsaturated structure of the molecule itself [29]. Although both the *cis* and *trans* forms of oleic acid are deemed unsaturated, it is important to understand that the inclusion of a *trans* double bond in a fatty acyl chain initiates a reduced bonding angle relative to a *cis* double bond, leading to a fatty acid acyl chain conformation that bears a substantial resemblance to a saturated fatty acid structure rather than an unsaturated fatty acid structure, regardless overall unsaturation [95].

On the other hand, clofazimine delivery enabled by ARG SEDDS is probably due to an increased stearic acid concentration as well as an increased viscosity. Stearic acid is a saturated fatty acid with a melting point of 69.3 °C that is significantly higher than skin surface temperature [51,96]. Consequently, stearic acid in its undissolved state, possibly provides a residual layer on the skin surface that enables improved occlusivity [26,97]. Occlusion, which is also established via the increased viscosity of a formulation, decreases transepidermal water loss and thus enhances swelling of the SC, which sequentially disrupts the strictly packed lipid structure of the SC rendering improved drug diffusion [98,99].

Finally, AVO2 displayed significantly (*p* < 0.018) lower permeation of the epidermis-dermis layer, probably due to the increased palmitic acid levels present within this oil [91,100]. Avocado oil contains higher concentrations saturated fatty acids compared to the other oils, particularly saturated fatty acids (e.g., palmitic and stearic acids) with longer carbon chain lengths (>C-12) that are known to be less effective penetration enhancers. The longer chain saturated fatty acids have a higher affinity toward the lipids within the SC as a result of the lipophilic nature and may therefore have delayed permeation of clofazimine into the skin due to hydrophobic interactions. Furthermore, the difference in lipid solubility and structure between saturated fatty acids and unsaturated fatty acids, may influence accumulation of clofazimine in the epidermis-dermis. Similar to stearic acid, palmitic acid has a melting point in the range of 31–59 °C, which is significantly higher than the melting points of unsaturated fatty acids (−50 to 4 °C). Thus, as stated, it is anticipated that these saturated fatty acids will display lower solubility and subsequently the avocado oil will be a semisolid at the temperatures at which the experiments were conducted. In addition, palmitic acid is of a linear shape, which lessened its ability to disrupt the lipid packing of the SC and to insert itself into the lipid bilayers, resulting in little or no effect(s) and prolonging the lag time [28].

As stated previously, drug delivery enabled by SEDDSs is fundamentally subjective to droplet size, droplet size distribution and zeta-potential; where a reduced droplet size normally meaningfully enhances rapid and increased drug permeation during dermal drug delivery [66]. However, with this study no direct correlation between dermal delivery and droplet size or zeta-potential could be recognized. Furthermore, no linear relationship between %EE or drug release and dermal diffusion was obtained. In a previous study performed by van Zyl et al. [3], where clofazimine diffusion through the skin was similarly tested either without it being combined into a drug delivery system, or where it was incorporated into liposomes, niosomes or transferosomes; we found that when included into transferosomes the highest concentrations of clofazimine, though only 2.18 μg/mL in the SC-epidermis and 0.73 μg/mL in the epidermis-dermis, could maximum be obtained. When no drug delivery system was employed, not even a mere 0.25 μg/mL clofazimine could be detected in either the SC-epidermis or epidermis-dermis. Comparing results acquired from this study, it is clear that incorporating clofazimine into SEDDS considerably increased drug permeability of the epidermis-epidermis as values between approximately 2.9–9.1 μg/mL, depending the type of natural oil incorporated, were measured.

### 3.3. Isothermal Microcalometry

The ARG and clofazimine combination rendered an average heat flow of 2.261 μW/g and an interaction error of 12.296 μW/g, signifying a small potential incompatibility. Potential hydrogen peroxide formation due to redox reactions facilitated by clofazimine might be responsible for these results [101,102,103,104,105]. This possible incompatibility detected may not even reflect a true incompatibility, but rather just confirm the presence of enhanced kinetic barriers within ARG SEDDSs as prolonged self-emulsification times were observed. 

Another explanation can be provided by the increased polyunsaturated fatty acid content of ARG (Supplementary Material). Polyunsaturated fatty acids are considered major oxidation targets due to lipid peroxidation reactions that lead to the disturbance of normal membrane structures [106]. Lipid peroxidation can be avoided by adding an antioxidative agent such as vitamin E [106]. It might therefore be beneficial to include anti-oxidative agent(s) in ARG SEDDs rather than focusing on the addition of oils containing saturated fatty acids that are less prone toward oxidation reactions. Unsaturated fatty acids are deliberated more powerful skin penetration enhancers compared to saturated fatty acids [27,101,102].

Potential incompatibilities were furthermore depicted between Span^®^60 and ARG (35.245 μW/g), MAC (48.270 μW/g) and OLV (33.930 μW/g), respectively. This was perhaps only due to physical interactions as Span^®^60, which does not easily cause skin irritations, is frequently included as a co-surfactant within topical formulations; often with Tween^®^80 [38]. Moreover, no incompatibilities between Span^®^60 and any oil have previously been reported. Nonetheless, other co-surfactants should also rather be investigated for inclusion into these SEDDSs. Detection of interactions should not dismay further investigation as incompatibilities can either be physical or chemical. Physical interactions between excipients are not exclusively unfavorable as it does not necessarily influence formulation stability [107].

### 3.4. Discussion

New scientific insights are essential to conduct intensified research for the purpose of developing new tools as alternatives to investing in the development of novel drug entities [108]. This study is in line with the “End-TB” strategy as it provides possible present-day solutions to an ancient disease by incorporating a repurposed drug into topical SEDDSs. Moreover, this study is a novel approach in the field of topical drug delivery by combining the knowledge gained during development of oral SEDDSs and applying it to conquer the formidable barrier provided by the SC. Dermal drug delivery is a challenging field since drugs should refrain from entering the systemic circulation while establishing a localized effect after crossing the SC [3]. In addition, the topical delivery of drugs as highly lipophilic as clofazimine presents unique challenges due to partitioning into, and accumulation in the SC post release from applied dosage forms; therefore, not being able to render a pharmacological effect.

Although not all intrinsic properties of clofazimine are considered suitable for dermal drug delivery; and despite potential incompatibilities that need to be further researched, SEDDSs comprising ARG, AVO, MAC or OLV rendered prominent dermal clofazimine transport due to the addition of natural oils known for their skin penetration enhancement properties; which are more affordable alternatives to certain chemical skin penetration enhancers. On the other hand, although all of the selected natural oils enhanced the solubilization of clofazimine, CCT formulations could not be deemed suitable topical clofazimine SEDDSs as all of these formulations displayed phase separation, indicating formulation instability. Interestingly, ARG3, Mac2 and OLV5 could be described as self-micro-emulsifying drug delivery systems (SMEDDSs), whereas AVO2 fell within the nano-range (SNEDDS). However, contrary to the literature [66], clofazimine delivery via the selected SEDDSs did not follow normal trends, where it has been postulated that drug delivery is fundamentally faster and increased when the droplet size and zeta-potential are reduced and viscosity is enhanced due to higher occlusivity. Rather, a decrease in the viscosity of the SEDDSs with an increase in the average droplet size was observed; which led to enhanced dermal delivery of clofazimine. It was furthermore found that a co-correlation exists between the pH of SEDDS and zeta-potential measurements as AVO SEDDS portrayed decreased pH values with increasingly negative zeta-potential values. Additionally, the ability to entrap clofazimine could not be linked to the oil or water content of the SEDDS formulations but as the droplet size and PDI values increased and the zeta potential decreased; the %EE was enhanced. Nonetheless, it could be deliberated that drug release was rather influenced by the oleic acid content of the different oils utilized. Overall, SEDDSs containing oils high in oleic acid released more clofazimine than oils rich in linoleic acid. Moreover, SEDDSs comprising oils high in stearic acid and an almost equal mixture of monounsaturated fatty acids and polyunsaturated fatty acids illustrated reduced clofazimine release. Similarly, it is reflected that fatty acid content, and thus, the type of natural oil included into the topical SEDDS, played a more noticeable role in clofazimine delivery into the skin than the physical characteristics of the SEDDSs as seen where OLV5 portrayed the highest clofazimine concentration in the epidermis-dermis.

In view of the characterization profiles of the selected topical SEDDSs, AVO2 was deliberated most suitable for topical clofazimine delivery as it falls within the nano-sized range; it exhibited the most negative zeta-potential value, signifying increased stability; it depicted the highest cloud point temperature, postulating dehydration of excipients at temperatures exceeding 40 °C; AVO2 received D-grading for self-emulsification efficacy (dull, greyish white appearance with an additional oily appearance together with slow emulsification ), predicting enhanced occlusive effects. Furthermore, finally, the pH of AVO2 is considered compatible with natural skin pH. However, AVO2 displayed the least favorable drug release characteristics. 

With regards to the criteria utilized during this study, it is deliberated necessary to redefine some of the specified standards, as these values should be more relatable to topical/transdermal SEDDS formulations. For example, PDI alone cannot dictate the suitability of topical/transdermal SEDDS. Microscopic techniques should be included and a particular range specified. The volume employed during the robustness to dilution experiment should also be adjusted as formulations intended for topical/transdermal delivery will never be exposed to such a high amount of dissolution medium. We furthermore suggest that the pH range for these formulations should mimic skin pH tolerability (4.5–5.0) and self-emulsification should, unlike during delivery of oral SEDDS, be slower during topical/transdermal drug delivery as diffusion through the lipophilic SC marks the rate limiting step for most drugs [29,79]. Consequently, extended contact time between SEDDSs and skin may enhance drug diffusion across the SC.

## 4. Conclusions

In order for topically applied SEDDSs to establish a localised pharmacological effect, they must reach the epidermis skin layer [29]. Topical delivery of the highly lipophilic clofazimine presented challenges due to the fact that drugs as hydrophobic as clofazimine tend to partition into and accumulate in the SC after being released from the applied dosage form [32,33]. The lipophilic nature of the SC creates an ideal environment for lipophilic drugs to establish a reservoir effect while these substances avoid full partitioning into the hydrophilic epidermis [32,43]. Hence, the rate limiting step for clofazimine is the partitioning into the hydrophilic epidermis [29]. For these reasons, it could be deliberated that the inclusion of clofazimine into topically applied SEDDSs was successful as all of the finally selected SEDDS formulations rendered delivery of clofazimine through the SC and into the epidermis-dermis skin layer. However, variances observed between the different SEDDS formulations and conclusive clofazimine delivery could not be directly related to droplet size, size distribution and zeta-potential; where a reduced droplet size and increased zeta-potential normally meaningfully enhance rapid and increased drug permeation during dermal drug delivery [66]. In this study, inverse correlations were obtained. Furthermore, no relationship between %EE or drug release and dermal diffusion was achieved. These factors may rather be linked to the unique composition of each plant-based oil [26]. Free fatty acids are naturally present within plant-based oils and are known skin penetration enhancers. Moreover, lipid disruption of the SC is established in many different ways as assisted by individual free fatty acids [26,27,28,29]. Monounsaturated fatty acids are considered more efficient skin penetration enhancers compared to saturated fatty acids as also displayed by the notably increased permeation of clofazimine [27]. The finally selected OLV5 comprising the highest oleic acid content rendered the most effective skin penetration enhancement properties during this study. Additionally, argan oil can be considered a useful skin penetration enhancer, despite decreased oleic acid content, as the possible occlusive effects of this plant-based oil established the second highest permeation characteristics of clofazimine during this research. Comparing our results to previous studies, it is clear that incorporating clofazimine into SEDDSs noticeably increased drug permeability of the epidermis-epidermis, depending the type of natural oil incorporated. 

To conclude, SEDDSs originally developed to enhance the oral drug delivery of lipophilic drugs can be considered a prospective topical and/or transdermal vehicle in terms of optimized dermal drug delivery, especially for drugs as lipophilic as clofazimine. However, although it was found that most of the criteria set for these types of oral drug delivery systems may be applied to topical SEDDSs, adjustments to some characterisation experiments—for example, robustness to dilution, PDI and pH—should be researched and described in more detail. The simplicity and ease of the preparation technique compared to methods followed to manufacture liposomes and nano-emulsions sets topical SEDDSs apart from current topical/transdermal drug delivery systems, especially when considering industrial upscaling and the possibility of individualized therapy. We would like to emphasize the potential of topical/transdermal SEDDSs to aid in diseases worsened by lymphatic dissemination, including Human Immunodeficiency Virus, metastatic cancers and endogenous extra-pulmonary TB, due to the lipid-based nature of SEDDSs potentially favorable for lymphatic uptake via topical application [108,109,110,111].

## Figures and Tables

**Table 1:** Grading system for emulsification behavior exhibited by SEDDSs upon dilution.

Grading	Description
Grade A	Swift emulsion formation, demonstrating a clear/bluish appearance (60 s)
Grade B	Swift formation of emulsion, which appears bluish (60 s)
Grade C	Emulsion displays fine milky appearance (120 s)
Grade D	Dull, greyish white appearance with an additional oily appearance together with slow emulsification, (>120 s)
Grade E	Poor or minimal emulsification noted with large oil droplets noticed on the surface

**Table 2:** Solubility of clofazimine determined in selected natural oils.

Natural Oil	Solubility of Clofazimine (mg/mL)
**Argan oil (ARG)**	2.23 ± 0.54
**Avocado oil (AVO)**	6.29 ± 0.44
**Coconut oil (CCT)**	1.78 ± 0.67
**Macadamia oil (MAC)**	1.25 ± 0.46
**Olive oil (OLV)**	1.09 ± 0.65

**Table 3:** Characterization profiles of topical SEDDSs that did not display phase separation after a period of 24 h at an ambient temperature. Characteristics that did not meet the criteria for the specific experiment conducted, are indicated in bold and highlighted.

SEDDS	Droplet Size (nm)	PDI	Zeta-Potential (mV)	Self-Emulsification Grading	Self-Emulsification Time (s)	Viscosity (mPa.s)	pH	Cloud Point (°C)
**ARG1**	66.24	0.39	−30.60	D	499	230.53	6.95	**27.00**
**ARG3**	107.32	0.62	−29.90	D	482	9436.27	6.00	34.00
**ARG5**	**440.78**	**1.00**	−23.40	D	423	1060.14	6.60	46.90
**AVO2**	64.11	0.34	−32.80	D	423	4103.20	5.01	40.00
**AVO3**	**345.75**	**1.00**	−40.90	D	131	710.40	**3.82**	45.90
**AVO4**	221.95	**1.00**	−37.50	D	130	6971.10	5.02	44.00
**AVO5**	59.013	0.55	−38.20	D	430	1178.40	5.05	50.60
**MAC1**	108.01	0.80	−25.00	D	374	23,071.00	7.40	**31.90**
**MAC2**	108.71	0.76	−29.20	D	346	17,476.53	6.61	32.50
**MAC3**	116.68	0.73	−27.70	D	181	5748.37	7.01	**31.40**
**MAC5**	**462.32**	**1.00**	−26.00	**B**	59	993.60	7.02	36.00
**OLV1**	**360.25**	**1.00**	−21.30	D	393	2511.03	7.37	34.80
**OLV2**	222.87	**1.00**	−30.00	D	385	6006.17	7.13	34.80
**OLV3**	**502.08**	**1.00**	−31.00	D	274	11,740.00	7.13	33.80
**OLV5**	154.80	0.80	−23.60	C	90	7220.00	6.97	35.90

**Table 4:** Data obtained for membrane release studies after 6 h. Average cumulative concentration is presented as mean ± standard error, whereas average percentage released is displayed as mean ± standard deviation.

Topical SEDDS Formulation	Average % Released	Average Cumulative Concentration (μg/mL)	Median of Cumulative Concentration (μg/mL)	Average % Released
**ARG3**	0.5 ± 0.002	0.729 ± 0.030	0.726	0.5 ± 0.002
**AVO2**	2.5 ± 0.007	1.685 ± 0.161	1.707	2.5 ± 0.007
**MAC2**	2 ± 0.002	0.586 ± 0.038	0.571	2 ± 0.002
**OLV5**	7 ± 0.002	0.459 ± 0.350	0.445	7 ± 0.002

## Supplementary Materials

The following are available online at https://www.mdpi.com/1999-4923/12/6/523/s1.
