# Supplementary material for: Development of a Self-Emulsifying Drug Delivery System for Optimized Topical Delivery of Clofazimine
Source: Pharmaceutics. 2020 Jun 8;12(6):523. doi: 10.3390/pharmaceutics12060523 (PMC7356627; doi:10.3390/pharmaceutics12060523)
Supplement: Supplementary file 1 [file pharmaceutics-12-00523-s001.pdf]

# SUPPLEMENTARY DATA:

# Section A:

## Fatty acid composition of selected plant based oils

The fatty acid composition of these natural oils is presented in Table 1A where the table was compiled from information obtained from different researched papers that focused on providing fatty acid profiles of natural oils.

**Table 1A.** Fatty acid composition of selected plant based oils (34–36).

| Fatty acid (% ± SD) | Argan oil | Avocado oil | Coconut oil | Macadamia oil | Olive oil |
|---|---|---|---|---|---|
| **Lauric acid** | Not detected | Not detected | **66.94 ± 4.11** | Not detected | Not detected |
| **Myristic acid** | 0.12 ± 0.01 | 0.01 ± 0.008 | **18.81 ± 0.98** | 0.91 ± 0.17 | Not detected |
| **Palmitic acid** | 11.80 ± 0.53 | **14.21 ± 1.02** | 6.54 ± 1.20 | 6.84 ± 1.87 | 0.73 ± 0.05 |
| **Palmitoleic acid** | 0.18 ± 0.02 | 7.06 ± 0.79 | Not detected | **9.68 ± 0.90** | 2.04 ± 0.03 |
| **Stearic acid** | **6.00 ± 0.05** | 2.15 ± 0.07 | 2.01 ± 0.12 | 3.39 ± 0.96 | 2.83 ± 0.01 |
| **Oleic acid** | 45.90 ± 1.84 | 59.46 ± 3.88 | 4.75 ± 0.1 | 50.65 ± 8.05 | **72.79 ± 0.21** |
| **Linoleic acid** | **34.10 ± 1.08** | 14.66 ± 1.20 | 0.82 ± 0.32 | 8.63 ± 1.77 | 13.20 ± 0.03 |
| **Linolenic acid** | 0.13 ± 0.01 | 1.3 ± 0.15 | Not detected | **14.59 ± 1.20** | 0.92 ± 0.06 |
| **SFAs*** | 17.58 ± 0.63 | 16.93 ± 1.09 | **94.40 ± 5.08** | 14.56 ± 2.38 | 11.60 ± 0.33 |
| **MUFAs*** | 46.08 ± 1.82 | 67.11 ± 1.77 | 4.78 ± 0.17 | 62.22 ± 9.19 | **73.52 ± 0.21** |
| **PUFAs*** | **33.63 ± 1.25** | 15.96 ± 1.15 | 0.082 ± 0.32 | 23.22 ± 1.62 | 14.13 ± 0.10 |
| **UFAs/SFAs** | 3.85 | 4.90 | 0.059 | **5.86** | 4.97 |

*** Monounsaturated Fatty Acids (MUFAs), Polyunsaturated Fatty Acids (PUFAs), Unsaturated Fatty Acids (UFAs), Saturated Fatty Acids (SFAs)**

## References


34. Cicero, N.; Albergamo, A.; Salvo, A.; Bua, G.D.; Bartolomeo, G.; Mangano, V.; Rotondo, A.; Di Stefano, V.; Di Bella, G.; Dugo, G. Chemical characterization of a variety of cold-pressed gourmet oils available on the Brazilian market. *Food Res Int*. **2018**, *109*, 517–525, doi: 10.1016/j.foodres.2018.04.064.

35. El Kharrassi, Y.; Maata, N.; Mazri, M.A.; El Kamouni, S.; Talbi, M.; El Kebbaj, R.; Moustaid, K.; Essamadi, A.K.; Andreoletti, P.; Cherkaoui-Malki, M.; Nasser, B. Chemical and phytochemical characterizations of argan oil (Argania spinosa L. skeels), olive oil (Olea europaea L. cv. Moroccan picholine), cactus pear (Opuntia megacantha salm-dyck) seed oil and cactus cladode essential oil. *J. Food Meas. Charact*. **2018**, *12*, 747-754, doi: 10.1007/s11694-017-9688-x.

36. Rueda, A.; Seiquer, I.; Olalla, M.; Giménez, R.; Lara, L.; Cabrera-Vique, C. Characterization of fatty acid profile of argan oil and other edible vegetable oils by gas chromatography and discriminant analysis. *J. Chem.* **2014**, *2014*, 843908, doi: 10.1155/2014/843908.

# Section B:

# SEDDS formulations selected from the various self-emulsification regions

A possible 5 formulations were prepared from each individual oil's self-emulsification region (Table 1B), i.e. two points at the top, two at the bottom, and one in the centre of the identified region on the pseudo-ternary phase diagrams (Figure 1 in paper). The self-emulsification area on the pseudo-ternary phase diagram of the coconut oil preparations rendered a markedly smaller range comparatively. Nonetheless, 5 possible formulations were selected from this region as similarly as possible to the selection method utilized for the other oils.

**Table 1B.** SEDDS formulations selected from the self-emulsification region of each individual oil where their respective abbreviations as well as the ratio of excipients are indicated. Formulations that depicted phase separation after 24 h, and thus considered inappropriate drug delivery systems, are highlighted and in bold. These formulations in bold were excluded from further analysis.

| Oil Type Included in SEDDS | Formulation Abbreviation | Excipient Ratio (Oil: Surfactant Phase: Water) |
|---|---|---|
| **Argan Oil (ARG)** | ARG1 | 7 : 5 : 7 |
| | **ARG2** | **7.4 : 5 : 5** |
| | ARG3 | 6 : 4 : 6 |
| | **ARG4** | **7 : 3 : 3** |
| | ARG5 | 4.4 : 3 : 7 |
| **Avocado Oil (AVO)** | **AVO1** | **7 : 5 : 5** |
| | AVO2 | 6.6 : 5 : 5 |
| | AVO3 | 6 : 4 : 6 |
| | AVO4 | 3.4 : 3 : 7 |
| | AVO5 | 7 : 3 : 7 |
| **Coconut Oil (CCT)** | **CCT1** | **9 : 3 : 7** |
| | **CCT2** | **9 : 2 : 8** |
| | **CCT3** | **8 : 2 : 8** |
| | **CCT4** | **9 : 1 : 9** |
| | **CCT5** | **6.3 : 1 : 9** |
| **Macadamia Oil (MAC)** | MAC1 | 7 : 5 : 5 |
| | MAC2 | 6.6 : 5 : 5 |
| | MAC3 | 6 : 4 : 6 |
| | **MAC4** | **7 : 3 : 7** |
| | MAC5 | 4.4 : 3 : 7 |
| **Olive Oil (OLV)** | OLV1 | 7 : 5 : 5 |
| | OLV2 | 6.7 : 5 : 5 |
| | OLV3 | 6 : 4 : 6 |
| | **OLV4** | **4.4 : 3 : 7** |
| | OLV5 | 4.4 : 3 : 7 |
